# Establishment of reference intervals for complete blood count in healthy adults at different altitudes on the Western Sichuan Plateau

**DOI:** 10.3389/fmed.2025.1586778

**Published:** 2025-05-21

**Authors:** QiaoLing Wang, JiaYu Liu, ShengLin Hu, JunWu Du, ShuZhi Zhou, ZhengLin Huang, Yanwu Liu, Kongjie Yang, Ling Zhang, Jian Yang, Qing Yuan

**Affiliations:** ^1^Hepatobiliary Surgery, Yaan People's Hospital, Ya'an, China; ^2^Oncology Surgery, Yaan People's Hospital, Ya'an, China; ^3^Department of Nursing, Yaan People's Hospital, Ya'an, China; ^4^Department of Anesthesia, Yaan People's Hospital, Ya'an, China; ^5^Department of Cardiology, Yaan People's Hospital, Ya'an, China; ^6^Department of Ultrasound, Yaan People's Hospital, Ya'an, China; ^7^Physical Examination Center, Yaan People's Hospital, Ya'an, China; ^8^Department of Science and Education, Yaan People's Hospital, Ya'an, China

**Keywords:** altitude, reference value, blood cell count, red blood cell count, white blood cell count, platelet count, hypoxia

## Abstract

**Background:**

A detailed reference range for complete blood count of healthy adults in the Western Sichuan Plateau region is unavailable. This study aimed to explore changes in complete blood count (CBC) in healthy adults across high-altitude regions of Ganzi Prefecture, Sichuan Province, to establish altitude-specific reference intervals and improve diagnostic accuracy and provide tailored clinical guidance for residents in these areas.

**Methods:**

CBC data from 3,485 healthy individuals from four altitudes—Guza (1,400 m), Lucheng (2,500 m), Luhuo (3,200 m), and Litang (4,100 m)—were collected between January 2022 and December 2023. The data were analyzed by sex, altitude, age groups, and their interactions to establish reference intervals.

**Results:**

CBC indicators showed significant changes with increasing altitude. Red blood cell (RBC) count, hemoglobin (HGB), hematocrit (HCT), white blood cell (WBC) count, and platelet (PLT) count all significantly increased as altitude rose (*p* < 0.05). Males had significantly higher RBC, WBC, and PLT counts than females across all altitudes (*p* < 0.05), except in Litang, where HCT and HGB differences between sexes were not significant (*p* < 0.05). With increasing age, RBC count, HCT, HGB, WBC count, and PLT count increased in both sexes. Most CBC indicators in the study population exceeded national reference intervals, except for HGB and HCT in males from Guza and Lucheng.

**Conclusion:**

This study established CBC reference intervals for the high-altitude regions of Ganzi Prefecture, revealing significant variations by altitude, sex, and age. These findings provide valuable insights for improving disease diagnosis and medical care for high-altitude residents.

## Introduction

The Western Sichuan Plateau, situated at the intersection of western Sichuan Providence and the eastern Tibet Autonomous Region, is a high-altitude region, with elevations typically exceeding 3,000 m and reaching over 5,000 m in some areas (1). This unique environment is characterized by a significant decrease in the partial pressure of atmospheric oxygen. Some studies have shown that in a plateau environment, for every 1,000 meters increase in altitude, the partial pressure of arterial oxygen decreases by an average of 1.60 kilopascals (2). Respectively, compared to low-altitude plain regions. Consequently, the region experiences thin air, reduced oxygen content, and unique climatic conditions that differ significantly from the low-altitude plains. At altitudes above 3,000 m, the body may experience hypoxic responses, including a steep oxygen dissociation curve and pronounced hypoxia (3, 4). High-altitude environments exert a significant impact on human physiological functions, particularly the blood system (5, 6).

In high-altitude areas, the hypoxic condition stimulates the human blood system to produce a series of compensatory reactions. Numerous studies have clearly shown that in order for the human body to adapt to the hypoxic environment at high altitudes, it often promotes an increase in red blood cell production. This process enhances the ability of red blood cell production and thus improves the oxygen-carrying function of the blood, which is a key adaptive change in the blood system when dealing with high-altitude environments. However, it is worth noting that this change is also accompanied by potential risks. Research has found that it can lead to an increase in the incidence of stroke (7). At the same time, the changes in blood components and status caused by the enhanced red blood cell production, such as an increase in blood viscosity, are likely to be associated with the incidence of ischemic heart disease and the prognosis of the condition (8). These phenomena fully highlight the depth and breadth of the impact of the high-altitude environment on the human blood system, revealing the complex and close connection between the high-altitude environment and human health. However, the internationally recognized reference intervals for complete blood count (CBC) are primarily derived from populations in low-altitude plain (9), which may lead to inaccuracies when applied to high-altitude residents (10, 11). Therefore, a standardized approach to assessing the CBC parameters of plateau residents is unsuitable (12). This study aimed to establish reference intervals tailored to high altitudes by systematically evaluating CBC indicators in healthy adults across different altitudes on the Western Sichuan Plateau.

## Materials and methods

### Study participants

From January 2022 to December 2023, relying on the four county towns of Guza Town (1,400 m), Lucheng Town (2,500 m), Luhuo County (3,200 m) and Litang County (4,100 m) in Garze Tibetan Autonomous Prefecture on the Western Sichuan Plateau, we made use of the sole local hospitals in each area, namely the Second People’s Hospital of Kangding City in Guza Town, Kangding People’s Hospital in Lucheng Town of Kangding City, Luhuo County People’s Hospital and Litang County People’s Hospital. Subsequently, we collected and conducted an in-depth analysis of the health examination data of the local residents. The inclusion criteria are as follows: (1) No inflammatory diseases within the past month, and no oral administration of antibiotics, anticancer drugs, or anticoagulants; (2) Body Mass Index (BMI) between 18.5 and 23.9; (3) The normal cardiopulmonary function was determined through electrocardiogram and chest fluoroscopy; (4) Normal liver and kidney function; (5) Age ≥18 years old; and (6) No diseases of the immune system or blood system. The exclusion criteria are as follows: (1) Suffering from inflammatory diseases within the past month, or currently taking oral antibiotics, anticancer drugs, or anticoagulants; (2) Body Mass Index (BMI) lower than 18.5 or higher than 23.9; (3) Abnormal cardiopulmonary function, such as suffering from coronary heart disease, chronic obstructive pulmonary disease, heart failure and other diseases affecting cardiopulmonary function; (4) Abnormal liver and kidney function, manifested as liver enzyme indexes exceeding the normal range, decreased glomerular filtration rate, elevated serum creatinine, etc.; (5) Age less than 18 years old; (6) Suffering from immune system diseases, such as systemic lupus erythematosus, rheumatoid arthritis, etc.; or blood system diseases, such as leukemia, anemia, thrombocytopenic purpura, etc.; (7) Having mental diseases and being unable to cooperate with the completion of the research-related processes and questionnaire filling; and (8) Women who are pregnant or breastfeeding. When determining the sample size, the formula *n* = (Z_1−*α*/2_+Z_1−*β*_)^2^σ^2^/δ^2^ was used to calculate the sample sizes for red blood cells (RBC), hematocrit (HCT), hemoglobin (HGB), white blood cells (WBC), and platelets (PLT) respectively. For the two-sided test, α was set at 0.05, Z_1−α/2_ was 1.96, the power (1−*β*) was set at 0.8, and Z_1−*β*_ was 0.84. The overall standard deviation *σ* and the minimum detectable difference *δ* for each index were set with reference to previous studies. After calculation, the required sample sizes for each index were obtained, and the maximum value among them, nHCT = 129, was taken as the basic sample size. Considering sample loss, the sample size was expanded by a proportion of 25%, and the planned sample size was approximately 161. After screening, the data of 3,485 participants were actually included (1,561 males and 1,924 females). The specific distribution is as follows: 521 participants (255 males and 266 females) from Guza Town; 980 participants (385 males and 595 females) from Lucheng Town; 821 participants (361 males and 488 females) from Luhuo County; and 1,135 participants (560 males and 575 females) from Litang County. Finally, a total of 3,485 participants (1,561 males and 1,924 females) were included in this study, which far exceeded the planned sample size and effectively ensured the reliability of the research results.

### Ethical statement

This study was approved by the Ethics Committee of the Ya’an City People’s Hospital (approval number: 2023 011). Written informed consent was obtained from all participants.

### Detection methods

A 2 mL venous blood sample was collected using a vacuum tube containing ethylenediaminetetraacetic acid dipotassium salt dihydrate (EDTA-K2) as the anticoagulant. CBC + DIFF analysis was performed using a Sysmex XN9000 (Sysmex, UKB, JPN) fully automated hematology analyzer with accompanying reagents calibrated using the provided calibration materials. All operations strictly adhere to standard operating procedures.

### Data processing and quality control

The test data were processed using Microsoft EXCEL (Microsoft, Redmond, Washington, United States). The parameters analyzed included white blood count (WBC), red blood count (RBC), hemoglobin (HGB) count, hematocrit (HCT) test, and platelet count (PLT). Differences across these indicators were assessed: sexes, altitudes, and age groups.

### Statistical analysis

Data analysis was performed using IBM SPSS Statistics for Windows, version 25.0 (IBM Corp., Armonk, NY, United States). Quantitative variables following a normal distribution were presented as mean ± standard deviation. A *t*-test of two independent samples was used to compare groups, with statistical significance set at *p < 0.05*.

## Results

### Effect of altitude on CBC parameters

In the study of the influence of altitude on human hematological indicators, we conducted an in-depth analysis of the complete blood count (CBC) indicators of populations in different altitude areas. The results showed a series of significant changes closely related to the altitude.

As the altitude gradually increased, the red blood cell count (RBC) of both males and females showed a clear upward trend. In Guza Town (1,400 m) at a low altitude, the average RBC value for males was 5.19 ± 0.97 × 10^12^/L, and for females it was 4.56 ± 0.78 × 10^12^/L. In Litang County (4,100 m) at a high altitude, the average RBC value for males increased to 6.08 ± 0.63 × 10^12^/L, and for females it increased to 5.23 ± 0.85 × 10^12^/L. The trends of changes in hematocrit (HCT) and hemoglobin (HGB) were similar to those of RBC, indicating that the human body improves its adaptability to the hypoxic environment by increasing the RBC-related indicators to ensure sufficient oxygen delivery to various tissues of the body.

Although the overall fluctuation range of the white blood cell count (WBC) was relatively small, there were also signs of changes with the increase in altitude. The differences in WBC values among different altitude areas were highly significant through statistical analysis (*F* = 68.656, *p* < 0.001), strongly suggesting that altitude has a non-negligible impact on the basic state of the human immune system. This impact may be related to complex physiological processes such as the generation and activity regulation of immune cells.

**Figure 1:**
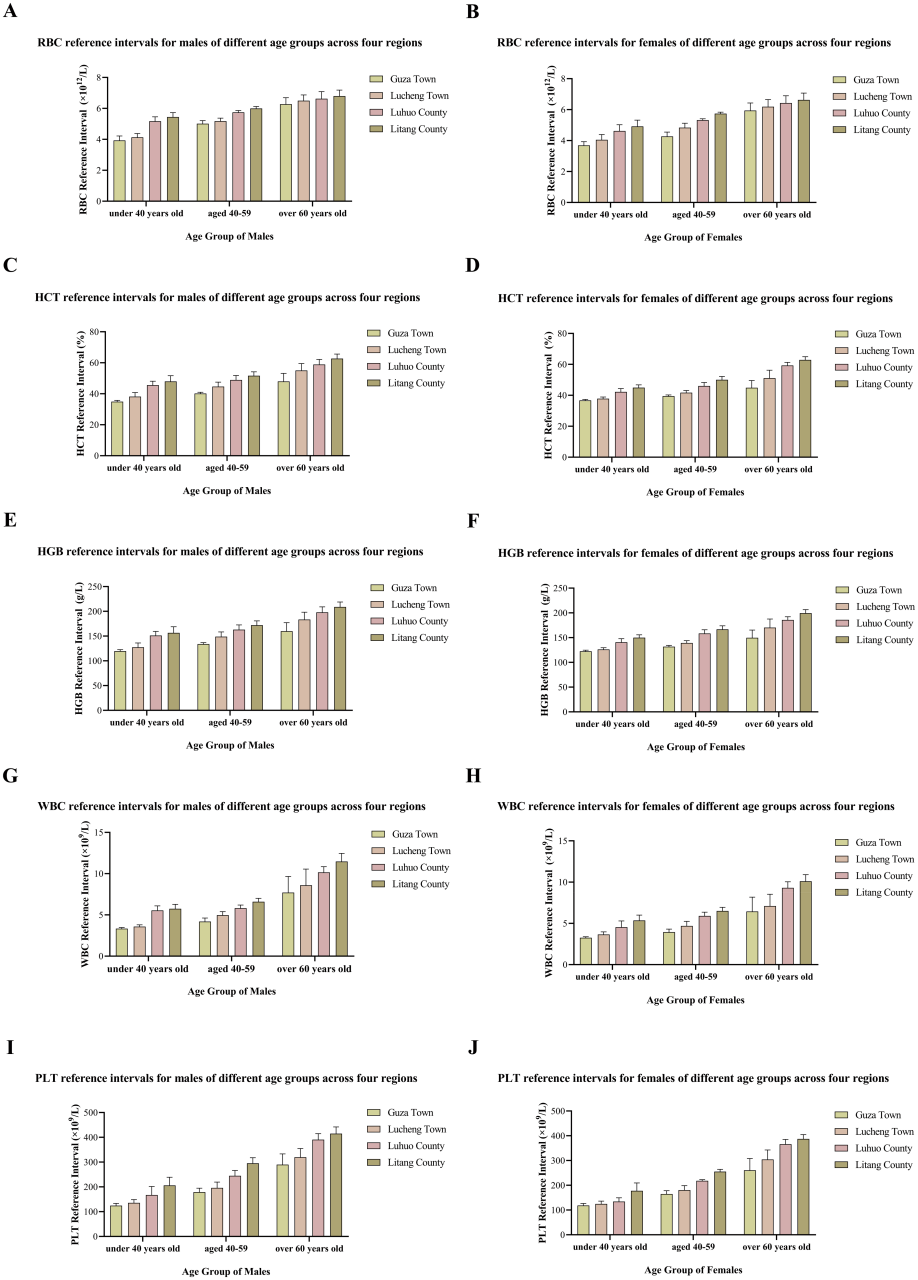
Graph showing the relationship between altitude and blood cells.

The platelet count (PLT) also increased with the increase in altitude. This change may be related to the adaptive adjustment of the body’s coagulation function in a high-altitude environment to cope with the increased risk of vascular damage that may be caused by factors such as hypoxia. The specific data are shown in Table 1 and Figure 1.

**Table 1:** Comparison of CBC indicators at different altitudes.

Indicator	WBC (×10^9^)	RBC (×10^12^)	HCT (%)	HGB (g/L)	PLT (×10^9^)
Male					
Guza Town (*n* = 255) (altitude 1400m)	5.24 ± 2.23	5.19 ± 0.97	42.27 ± 5.45	140.95 ± 18.16	207.73 ± 70.03
Lucheng Town (*n* = 385) (altitude 2500m)	6.40 ± 2.55	5.35 ± 0.80	44.46 ± 7.32	151.54 ± 14.38	245.61 ± 73.98
Luhuo County (*n* = 361) (altitude 3200m)	6.66 ± 1.72	5.50 ± 0.50	48.74 ± 4.97	162.47 ± 16.56	287.43 ± 59.37
Litang County (*n* = 560) (altitude 4100m)	7.52 ± 2.05	6.08 ± 0.63	53.77 ± 7.35	179.25 ± 24.49	305.63 ± 90.62
*F*	68.656	108.211	186.820	186.462	119.762
*P*	<0.001	<0.001	<0.001	<0.001	<0.001
Female					
Guza Town (*n* = 266) (altitude 1400m)	4.62 ± 1.70	4.91 ± 0.93	40.65 ± 4.30	135.56 ± 14.31	186.09 ± 63.95
Lucheng Town (*n* = 595) (altitude 2500m)	5.20 ± 1.48	5.09 ± 0.79	43.84 ± 5.43	146.12 ± 18.11	196.18 ± 66.93
Luhuo County (*n* = 488) (altitude 3200m)	5.62 ± 1.58	5.28 ± 0.62	45.58 ± 5.86	151.94 ± 19.53	206 ± 79.17
Litang County (*n* = 575) (altitude 4100m)	7.08 ± 2.64	5.70 ± 0.72	53.25 ± 8.01	177.51 ± 26.69	280.29 ± 91.32
F	135.421	94.233	336.298	335.926	172.773
P	<0.001	<0.001	<0.001	<0.001	<0.001

WBC, white blood cell count; RBC, red blood cell count; HCT, hematocrit; HGB, hemoglobin; PLT, platelets.

### Sex-based differences in CBC parameters

Regarding gender differences, the results showed diverse characteristics. In Guza Town (1,400 m), the mean value of white blood cell count (WBC) in males was 5.24 ± 2.23 × 10^9^/L, and in females it was 4.62 ± 1.70 × 10^9^/L. According to the *t*-test, the *t*-value was 3.36, and *p* < 0.001, with an extremely significant difference, indicating that the level of white blood cell count in males was relatively higher. In terms of red blood cell count (RBC), the mean value in males was 5.19 ± 0.97 × 10^12^/L, and in females it was 4.91 ± 0.93 × 10^12^/L. The *t*-value was 3.398, and *p* = 0.001. The RBC value in males was significantly higher than that in females. There were also significant gender differences in indicators such as hematocrit (HCT), hemoglobin (HGB), and platelet count (PLT) (all *p* < 0.001).

The situation in Lucheng Town (2,500 m) was similar. The mean value of WBC in males was 6.40 ± 2.55 × 10^9^/L, and in females it was 5.20 ± 1.48 × 10^9^/L. The *t*-value reached 10.183, and *p* < 0.001, with an extremely significant difference. In indicators such as RBC, HCT, HGB, and PLT, the values in males were generally higher than those in females, and the differences were highly significant according to statistical tests (all *p* < 0.001).

In Luhuo County (3,200 m), significant differences were also shown between males and females in various CBC indicators. The mean value of RBC in males was 5.50 ± 0.50 × 10^12^/L, and in females it was 5.28 ± 0.62 × 10^12^/L. The *t*-value was 3.834, and *p* < 0.001. For other indicators such as HCT, HGB, WBC, and PLT, there were also statistically significant gender differences (all *p* < 0.001).

The situation of gender differences in CBC indicators in Litang County (4,100 m) was slightly different. In terms of white blood cell count (WBC), the mean value in males was 7.52 ± 2.05 × 10^9^/L, and in females it was 7.08 ± 2.64 × 10^9^/L. The *t*-value was 3.147, and *p* = 0.002, with a significant difference. In red blood cell count (RBC), the value in males was 6.08 ± 0.63 × 10^12^/L, and in females it was 5.70 ± 0.72 × 10^12^/L. The *t*-value was 9.303, and *p* < 0.001, with a significant difference. In platelet count (PLT), the value in males was 305.63 ± 90.62 × 10^9^/L, and in females it was 280.29 ± 91.32 × 10^9^/L. The *t*-value was 8.696, and *p* < 0.001, with a significant difference. However, there were no significant differences in hematocrit (HCT) and hemoglobin (HGB) between genders (*p* = 0.254).

Overall, in most regions, there were significant differences between males and females in many CBC indicators, and the values of some indicators showed a trend of being higher in males than in females. However, the HCT and HGB indicators in Litang County (4,100 m) were exceptions. The specific data are shown in Table 2 and Figure 1.

**Table 2:** Comparison of CBC indicators between male and female residents in different altitudes.

Indicator	WBC (×10^9^)	RBC (×10^12^)	HCT (%)	HGB (g/L)	PLT (×10^9^)
Guza Town (altitude 1,400m)					
Male (*n* = 255)	5.24 ± 2.23	5.19 ± 0.97	42.27 ± 5.45	140.95 ± 18.16	207.73 ± 70.03
Female (*n* = 266)	4.62 ± 1.70	4.91 ± 0.93	40.65 ± 4.30	135.56 ± 14.31	186.09 ± 63.95
*t*	3.536	3.398	3.750	3.752	3.679
*P*	<0.001	0.001	<0.001	<0.001	0.001
Lucheng Town (altitude 2,500m)					
Male (*n* = 385)	6.40 ± 2.55	5.35 ± 0.80	44.46 ± 7.32	151.54 ± 14.38	245.61 ± 73.98
Female (*n* = 595)	5.20 ± 1.48	5.20 ± 1.48	43.84 ± 5.43	146.12 ± 18.11	196.18 ± 66.93
*t*	10.183	8.889	10.652	10.652	8.456
*P*	<0.001	<0.001	<0.001	<0.001	<0.001
Luhuo County (altitude 3,200m)					
Male (*n* = 361)	6.66 ± 1.72	5.50 ± 0.50	48.74 ± 4.97	162.47 ± 16.56	287.43 ± 59.37
Female (*n* = 488)	5.62 ± 1.58	5.28 ± 0.62	45.58 ± 5.86	151.94 ± 19.53	206 ± 79.17
*t*	6.798	5.854	8.487	8.487	11.148
*P*	<0.001	<0.001	<0.001	<0.001	<0.001
Litang County (altitude 4,100m)					
Male (*n* = 560)	7.52 ± 2.05	6.08 ± 0.63	53.77 ± 7.35	179.25 ± 24.49	305.63 ± 90.62
Female (*n* = 575)	7.08 ± 2.64	5.70 ± 0.72	53.25 ± 8.01	177.51 ± 26.69	280.29 ± 91.32
*t*	3.147	9.305	1.141	1.141	4.690
*P*	0.002	<0.001	0.254	0.254	<0.001

WBC, white blood cell count; RBC, red blood cell count; HCT, hematocrit; HGB, hemoglobin; PLT, platelets.

### Age-based comparison of CBC indicators

In Litang County, there was a clear association between age increase and the rise of multiple CBC indicators. For the male population, when the age was <40 years old, the mean value of white blood cell count (WBC) was 5.74 ± 0.54 × 10^9^/L, and the mean value of red blood cell count (RBC) was 5.44 ± 0.28 × 10^12^/L. When the age reached 60 + years old, the WBC increased to 11.47 ± 0.99 × 10^9^/L, and the RBC increased to 6.80 ± 0.38 × 10^12^/L. A similar trend was also observed in the female population. The mean value of RBC at 60 + years old was 6.63 ± 0.44 × 10^12^/L, which was higher than 4.92 ± 0.40 × 10^12^/L at <40 years old.

The situation in Luhuo County was similar. For males <40 years old, the mean value of WBC was 5.55 ± 0.54 × 10^9^/L, and it increased to 10.16 ± 0.69 × 10^9^/L at 60+ years old. In the female population of the corresponding age-groups, the RBC increased from 4.62 ± 0.40 × 10^12^/L (<40 years old) to 6.43 ± 0.47 × 10^12^/L (60 + years old).

Lucheng Town and Guza Town also demonstrated the correlation between age and CBC indicators. In Lucheng Town, the mean value of HGB for males at 60 + years old was 183.35 ± 14.85g/L, which was higher than 148.87 ± 9.54g/L for those aged 40–59 years old. In Guza Town, the mean value of PLT for males at 60 + years old was 289.70 ± 43.62 ×10^9^/L, which was higher than 178.87 ± 15.72 × 10^9^/L for those aged 40–59 years old.

In addition, within the same age-group, gender differences in CBC indicators were also quite significant in various regions. For example, in the age-group <40 years old in Litang County, the mean value of WBC in males was higher than that in females. In the age-group <40 years old in Guza Town, the mean value of HCT in females was higher than that in males.

In conclusion, the CBC indicators of residents in various regions were positively correlated with age, and the gender factor also had a significant impact on CBC indicators within the same age-group. The specific data are shown in Table 3 and Figure 1.

**Table 3:** Comparison of CBC indicators among residents of different age groups in different altitudes.

Indicator		WBC (×10^9^)	RBC (×10^12^)	HCT (%)	HGB (g/L)	PLT (×10^9^)
Guza Town (altitude 1,400m)						
Ages (years) < 40	Male (*n* = 64)	3.33 ± 0.14	3.93 ± 0.29	34.88 ± 0.9	119.67 ± 2.99	124.56 ± 8.94
	Female (*n* = 66)	3.25 ± 0.13	3.69 ± 0.24	36.67 ± 0.72	122.31 ± 2.4	118.50 ± 8.10
40–59	Male (*n* = 99)	4.19 ± 0.43	5.01 ± 0.20	40.14 ± 0.85	133.87 ± 2.83	178.87 ± 15.72
	Female (*n* = 109)	3.94 ± 0.37	4.27 ± 0.28	39.50 ± 0.75	131.72 ± 2.48	164.88 ± 13.4
60+	Male (*n* = 92)	7.70 ± 1.96	6.27 ± 0.42	47.96 ± 5.21	159.89 ± 17.36	289.70 ± 43.62
	Female (*n* = 91)	6.44 ± 1.74	5.94 ± 0.49	44.92 ± 4.65	149.77 ± 15.51	260.53 ± 47.74
Lucheng Town (altitude 2,500m)						
Ages (years) < 40	Male (*n* = 34)	3.58 ± 0.21	4.13 ± 0.25	38.17 ± 2.61	127.41 ± 8.69	135.79 ± 12.76
	Female (*n* = 80)	3.65 ± 0.32	4.05 ± 0.34	37.86 ± 1.05	126.2 ± 3.5	124.96 ± 10.99
40–59	Male (*n* = 180)	4.97 ± 0.42	5.18 ± 0.19	44.66 ± 2.86	148.87 ± 9.54	196.01 ± 23.43
	Female (*n* = 356)	4.69 ± 0.55	4.84 ± 0.28	41.68 ± 1.40	139.26 ± 4.67	180.48 ± 18.23
60+	Male (*n* = 171)	8.60 ± 1.96	6.50 ± 0.37	55.01 ± 4.46	183.35 ± 14.85	319.65 ± 34.96
	Female (*n* = 159)	7.11 ± 1.42	6.19 ± 0.46	51.12 ± 5.14	170.38 ± 17.14	304.57 ± 38.27
Luhuo County (altitude 3,200m)						
Ages (years) < 40	Male (*n* = 200)	5.55 ± 0.54	5.17 ± 0.29	45.57 ± 2.50	151.23 ± 8.35	167.32 ± 34.04
	Female (*n* = 302)	4.55 ± 0.74	4.62 ± 0.40	42.21 ± 2.15	140.69 ± 7.17	134.4 ± 15.12
40–59	Male (*n* = 128)	5.82 ± 0.37	5.74 ± 0.12	48.88 ± 2.91	162.94 ± 9.70	244.98 ± 20.98
	Female (*n* = 130)	5.90 ± 0.45	5.32 ± 0.09	46.02 ± 2.27	158.39 ± 7.58	218.05 ± 4.46
60+	Male (*n* = 33)	10.16 ± 0.69	6.62 ± 0.46	58.87 ± 3.32	198.08 ± 11.06	390.70 ± 24.03
	Female (*n* = 56)	9.31 ± 0.73	6.43 ± 0.47	59.29 ± 1.99	185.63 ± 6.62	366.18 ± 18.84
Litang County (altitude 4,100m)						
Ages (years) < 40	Male (*n* = 190)	5.74 ± 0.54	5.44 ± 0.28	47.94 ± 3.69	156.46 ± 12.30	206.24 ± 32.75
	Female (*n* = 184)	5.36 ± 0.64	4.92 ± 0.40	44.98 ± 1.76	149.93 ± 5.86	177.77 ± 31.65
40–59	Male (*n* = 180)	6.59 ± 0.42	6.00 ± 0.12	51.60 ± 2.59	172.02 ± 8.62	295.31 ± 22.21
	Female (*n* = 174)	6.50 ± 0.44	5.74 ± 0.10	50.01 ± 2.16	166.70 ± 7.19	255.4 ± 8.93
60+	Male (*n* = 190)	11.47 ± 0.99	6.80 ± 0.38	62.67 ± 2.96	208.89 ± 9.86	414.78 ± 27.2
	Female (*n* = 217)	10.11 ± 0.8	6.63 ± 0.44	62.87 ± 2.08	199.57 ± 6.94	387.18 ± 17.78

WBC, white blood cell count; RBC, red blood cell count; HCT, hematocrit; HGB, hemoglobin; PLT, platelets.

### Sex and altitude-based reference intervals for CBC in residents of the Western Sichuan Plateau

From a gender perspective, there were significant differences in the reference intervals of CBC indicators between males and females. Taking the white blood cell count (WBC) as an example, the reference interval for males in Guza Town was (3.00, 9.51) × 10^9^/L, while for females it was (2.92, 8.09) × 10^9^/L. In terms of red blood cell count (RBC), the reference interval for males in Lucheng Town was (4.10, 6.74) × 10^12^/L, and for females it was (3.84, 6.49) × 10^12^/L. For most indicators, the upper limit of the reference interval for males was higher than that for females.

Regarding regional differences, as the altitude increased, the reference intervals of some indicators showed an upward trend. Comparing Litang County with Guza Town, for males, the lower limit of the red blood cell count (RBC) reference interval increased from 3.79 × 10^12^/L to 5.17 × 10^12^/L, and the upper limit increased from 6.63 × 10^12^/L to 7.13 × 10^12^/L. The lower limit of the hematocrit (HCT) reference interval increased from 36.04 to 44.07%, and the upper limit increased from 52.95 to 64.80%. This indicates that residents in high-altitude areas may have elevated blood oxygen-carrying related indicators as an adaptation to the hypoxic environment.

Compared with the national reference values, the reference intervals of some indicators in various regions deviated from them. For example, the lower limit of the red blood cell count (RBC) reference interval for males in Luhuo County was 4.80 × 10^12^/L, higher than the national reference value lower limit of 4.3 × 10^12^/L. The upper limit of the hematocrit (HCT) reference interval for males in Litang County was 64.80%, higher than the national reference value upper limit of 50%. The specific data are shown in Table 4 and Figure 1.

**Table 4:** Reference intervals complete blood count indicators in different regions.

Indicator	WBC (×10^9^)	RBC (×10^12^)	HCT (%)	HGB (g/L)	PLT (×10^9^)
Male					
Guza Town (*n* = 255) (altitude 1,400m)	(3.00, 9.51)	(3.79, 6.63)	(36.04, 52.95)	(115.81, 176.54)	(127.37, 326.48)
Lucheng Town (*n* = 385) (altitude 2,500m)	(3.51, 10.32)	(4.10, 6.74)	(36.35, 57.77)	(128.02, 189.19)	(141.21, 353.88)
Luhuo County (*n* = 361) (altitude 3,200m)	(4.72, 10.48)	(4.80, 6.77)	(41.72, 60.80)	(138.38, 199.65)	(179.33, 392.3)
Litang County (*n* = 560) (altitude 4,100m)	(5.08, 11.17)	(5.17, 7.13)	(44.07, 64.80)	(143.56, 215.98)	(173.68, 432.17)
National reference values	(3.5, 9.5)	(4.3, 5.8)	(40, 50)	(130, 175)	(125, 350)
Female					
Guza Town (*n* = 266) (altitude 1,400m)	(2.92, 8.09)	(3.60, 6.37)	(34.99, 49.49)	(119.43, 165.02)	(112.38, 302.07)
Lucheng Town (*n* = 595) (altitude 2,500m)	(3.37, 8.29)	(3.84, 6.49)	(37.11, 55.23)	(123.68, 184.10)	(116.34, 321.35)
Luhuo County (*n* = 488) (altitude 3,200m)	(3.92, 8.97)	(4.32, 6.63)	(38.86, 57.32)	(129.53, 191.04)	(115.80, 349.14)
Litang County (*n* = 575) (altitude 4,100m)	(4.40, 10.70)	(4.56, 6.93)	(43.39, 64.11)	(144.63, 213.69)	(163.71, 396.82)
National reference values	(3.5, 9.5)	(3.8, 5.1)	(35, 45)	(115, 150)	(125, 350)

CBC, complete blood count; WBC, white blood cell count; RBC, red blood cell count; HCT, hematocrit; HGB, hemoglobin; PLT, platelets.

### Age, sex, and altitude-specific CBC reference intervals in the Western Sichuan Plateau

From the perspective of age, as age increases, the reference intervals of multiple CBC indicators for residents in various regions show changes. Take the males in Litang County as an example. The reference interval of white blood cell count (WBC) is (4.68, 6.80) × 10^9^/L when the age is < 40 years old, and expands to (10.06, 12.76) × 10^9^/L at 60 + years old. The lower limit of the reference interval of red blood cell count (RBC) increases from 4.89 × 1,012/L at < 40 years old to 6.06 × 1,012/L at 60+ years old, and the upper limit increases from 5.99 × 1,012/L to 7.54 × 1,012/L. This kind of change reflects the physiological adjustment of the human blood system with age.

Regional differences are also significant. Comparing Lucheng Town at a low altitude and Litang County at a high altitude, the upper limits of the reference intervals of red blood cell count (RBC), hematocrit (HCT), and hemoglobin (HGB) for residents in Litang County in each age-group are generally higher. For example, the HCT reference interval for males aged 40–59 in Litang County is (46.52, 56.68)%, while for males of the same age-group in Lucheng Town it is (39.05, 50.27)%, indicating that for residents in high-altitude areas, the reference intervals of blood oxygen-carrying related indicators shift upward to adapt to the hypoxic environment.

**Figure 2:**
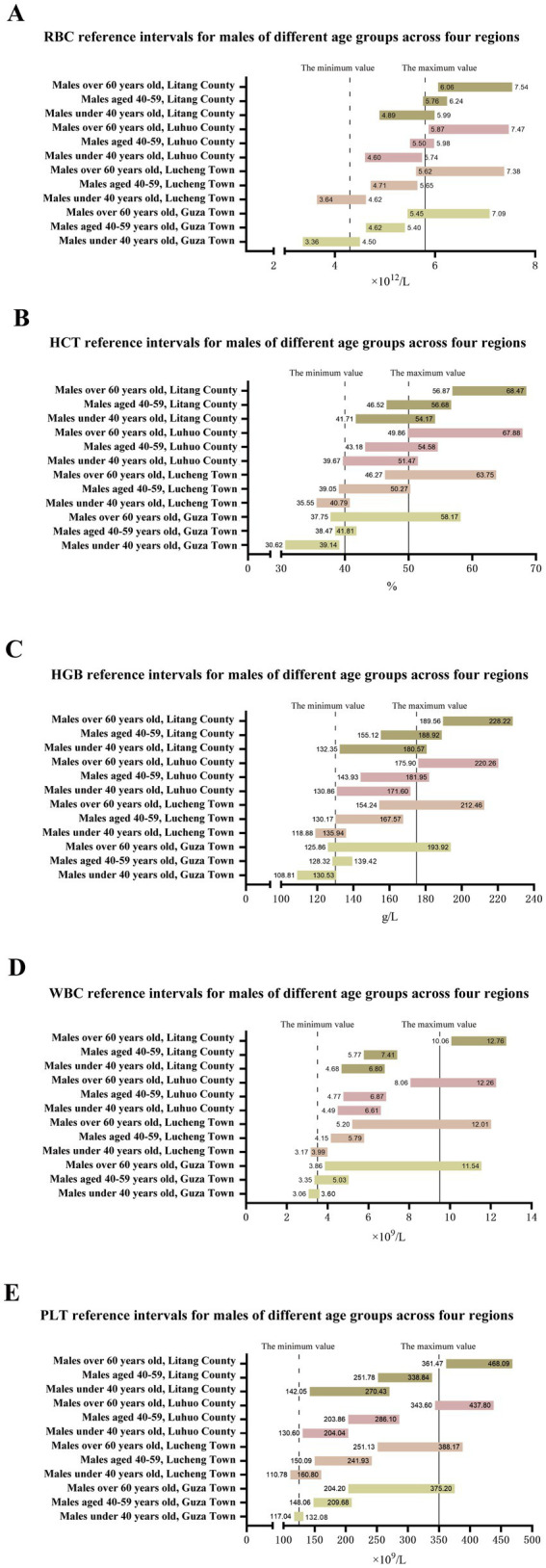
Reference interval of a complete blood count of male residents in different regions.

**Figure 3:**
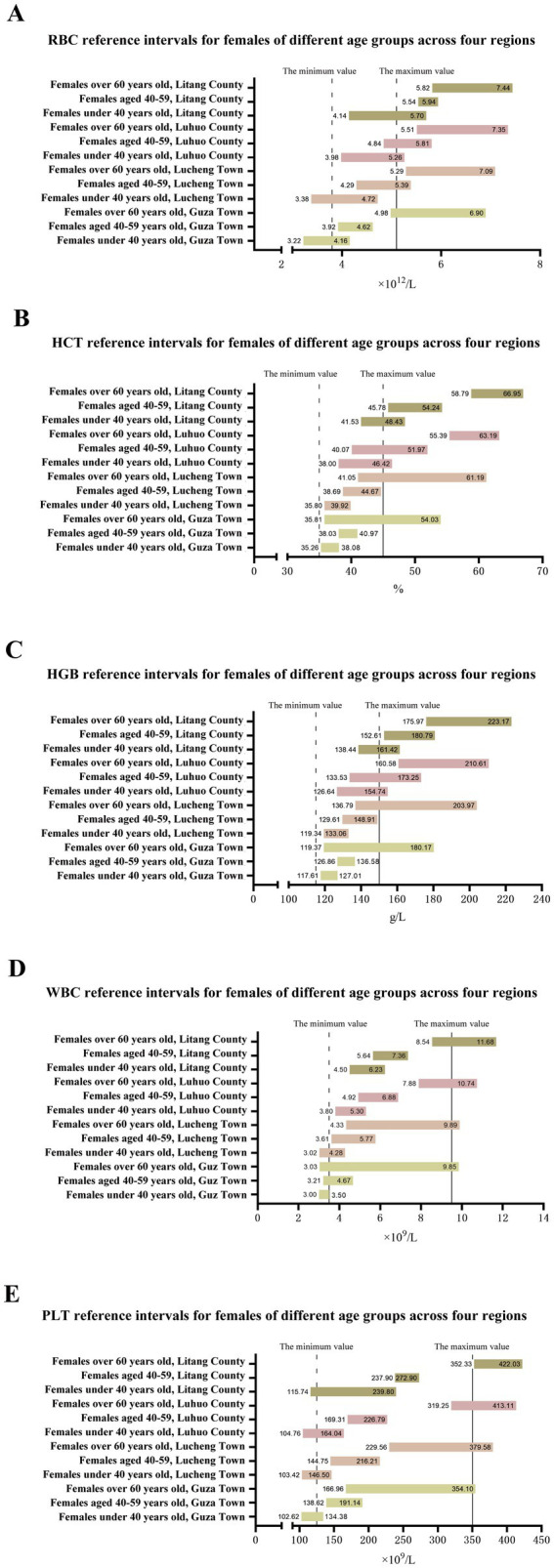
Reference interval of a complete blood count of female residents in different regions.

Compared with the national reference values, the reference intervals of CBC indicators for different age-groups in various regions deviate. For example, the lower limit of the RBC reference interval for males aged 60 + in Luhuo County is 5.87 × 1,012/L, which is higher than the national male reference value lower limit of 4.3 × 1,012/L. The upper limit of the HGB reference interval for females < 40 years old in Guza Town is 127.01g/L, which is lower than the national female reference value upper limit of 150g/L. The specific data are shown in Table 5 and Figures 2, 3.

**Table 5:** Reference intervals of a complete blood count for residents of different age groups in different regions.

Indicator		WBC (×10^9^)	RBC (×10^12^)	HCT (%)	HGB (g/L)	PLT (×10^9^)
Guza Town (altitude 1,400m)						
Ages (years) < 40	Male (*n* = 64)	(3.06, 3.60)	(3.36, 4.50)	(30.62, 39.14)	(108.81, 130.53)	(117.04, 132.08)
	Female (*n* = 66)	(3.00, 3.50)	(3.22, 4.16)	(35.26, 38.08)	(117.61, 127.01)	(102.62, 134.38)
40–59	Male (*n* = 99)	(3.35, 5.03)	(4.62, 5.40)	(38.47, 41.81)	(128.32, 139.42)	(148.06, 209.68)
	Female (*n* = 109)	(3.21, 4.67)	(3.92, 4.62)	(38.03, 40.97)	(126.86, 136.58)	(138.62, 191.14)
60+	Male (*n* = 92)	(3.86, 11.54)	(5.45, 7.09)	(37.75, 58.17)	(125.86, 193.92)	(204.2, 375.2)
	Female (*n* = 91)	(3.03, 9.85)	(4.98, 6.90)	(35.81, 54.03)	(119.37, 180.17)	(166.96, 354.10)
Lucheng Town (altitude 2,500m)						
Ages (years) < 40	Male (*n* = 34)	(3.17, 3.99)	(3.64, 4.62)	(35.55, 40.79)	(118.88, 135.94)	(110.78, 160.80)
	Female (*n* = 80)	(3.02, 4.28)	(3.38, 4.72)	(35.80, 39.92)	(119.34, 133.06)	(103.42, 146.5)
40–59	Male (*n* = 180)	(4.15, 5.79)	(4.71, 5.65)	(39.05, 50.27)	(130.17, 167.57)	(150.09, 241.93)
	Female (*n* = 356)	(3.61, 5.77)	(4.29, 5.39)	(38.69, 44.67)	(129.61, 148.91)	(144.75, 216.21)
60+	Male (*n* = 171)	(5.20, 12.01)	(5.62, 7.38)	(46.27, 63.75)	(154.24, 212.46)	(251.13, 388.17)
	Female (*n* = 159)	(4.33, 9.89)	(5.29, 7.09)	(41.05, 61.19)	(136.79, 203.97)	(229.56, 379.58)
Luhuo County (altitude 3,200m)						
Ages (years) < 40	Male (*n* = 200)	(4.49, 6.61)	(4.60, 5.74)	(39.67, 51.47)	(130.86, 171.6)	(130.60, 204.04)
	Female (*n* = 302)	(3.80, 5.30)	(3.98, 5.26)	(38.00, 46.42)	(126.64, 154.74)	(104.76, 164.04)
40–59	Male (*n* = 128)	(4.77, 6.87)	(5.50, 5.98)	(43.18, 54.58)	(143.93, 181.95)	(203.86, 286.1)
	Female (*n* = 130)	(4.92, 6.88)	(4.84, 5.81)	(40.07, 51.97)	(133.53, 173.25)	(169.31, 226.79)
60+	Male (*n* = 33)	(8.06, 12.26)	(5.87, 7.47)	(49.86, 67.88)	(175.9, 220.26)	(343.6, 437.8)
	Female (*n* = 56)	(7.88, 10.74)	(7.88, 10.74)	(5.51, 7.35)	(55.39, 63.19)	(319.25, 413.11)
Litang County (altitude 4,100m)						
Ages (years) < 40	Male (*n* = 190)	(4.68, 6.80)	(4.89, 5.99)	(41.71, 54.17)	(132.35, 180.57)	(142.05, 270.43)
	Female (*n* = 184)	(4.50, 6.23)	(4.14, 5.70)	(41.53, 48.43)	(138.44, 161.42)	(115.74, 239.8)
40–59	Male (*n* = 180)	(5.77, 7.41)	(5.76, 6.24)	(46.52, 56.68)	(155.12, 188.92)	(251.78, 338.84)
	Female (*n* = 174)	(5.64, 7.36)	(5.54, 5.94)	(45.78, 54.24)	(152.61, 180.79)	(237.90, 272.90)
60+	Male (*n* = 190)	(10.06, 12.76)	(6.06, 7.54)	(56.87, 68.47)	(189.56, 228.22)	(361.47, 468.09)
	Female (*n* = 217)	(8.54, 11.68)	(5.82, 7.44)	(58.79, 66.95)	(175.97, 223.17)	(352.33, 422.03)
National reference values		(3.5, 9.5)	Male (4.3, 5.8) Female (3.8, 5.1)	Male (40, 50) Female (35, 45)	Male (130, 175) Female (115, 150)	(125, 350)

CBC, complete blood count; WBC, white blood cell count; RBC, red blood cell count; HCT, hematocrit; HGB, hemoglobin; PLT, platelets.

## Discussion

Complete blood count (CBC) is an essential clinical test that evaluates an individual’s health by analyzing various blood components (13). As a simple, quick, and cost-effective examination test, the CBC is indispensable in clinical diagnosis, treatment monitoring, and health management (14). Altitude, sex, and age can significantly influence CBC results (15). Reference standards derived from low-altitude populations may not adequately represent populations residing at high altitudes (16, 17). Therefore, it is necessary to establish CBC reference intervals tailored to high-altitude regions to provide scientifically accurate and contextually relevant reference data for indigenous populations in these regions (18). Our findings reveal significant variability in CBC reference intervals across sex, age, and altitude. Certain CBC indicators exhibit sex-based discrepancies due to differences in physiological structure, hormone regulations, and lifestyle between men and women (19, 20). Moreover, age is a critical determinant of variation in CBC indicators (20, 21). Table 4 demonstrate that when age is not considered, CBC reference intervals for residents of different sexes largely overlap with the national standards. However, incorporating age stratification reveals pronounced differences in reference intervals for CBC indicators across age groups of residents in high-altitude areas. This emphasizes the necessity for accounting for sex and age when establishing CBC reference values for populations in high-altitude regions (9, 22).

Red blood cells are crucial CBC components primarily responsible for oxygen transport (23). RBC function is assessed through these parameters in RBC count, HCT, and HGB (24). HGB is an iron-containing protein found in red blood cells (25). In high-altitude environments, an increase in these indicators can be attributed to physiological adaptations and environmental stress (26). Reduced atmospheric oxygen pressure at high altitudes induces a relatively hypoxic state in the body. In response, hypoxia stimulates the kidneys to secrete erythropoietin (EPO) (27, 28), which then acts on the bone marrow to promote the production and proliferation of red blood cells, ultimately increasing the red blood cell count (29). As red blood cells increase, HCT and HGB levels increase (25, 30). The body requires higher oxygen delivery in hypoxic high altitudes environments to maintain normal physiological functions, as an increase in RBC, HCT, and HGB levels improves the oxygen-carrying capacity of the blood. This increases the amount of oxygen bound per unit volume of blood, thereby increasing the oxygen transport efficiency to meet the body’s oxygen demands. This phenomenon has been confirmed through animal studies (31). Further research has shown that mRNA expression levels of EPAS1 and PPARA in indigenous Tibetan populations living at high altitudes are significantly higher than in Han populations from low-altitude plains. This correlates with the higher hemoglobin levels observed in Tibetans, suggesting that genetic factors play a key role in adaptation to high-altitude environments (32, 33). As previous research has shown, a new gene, FBN1, has been found in at least 5% of the population living at high altitudes in Peru. Moreover, this gene is widely present among the people residing in the Andean Mountains region (34). However, relevant studies have also clearly pointed out that even among people of the same ethnic group, differences in the altitude at which they live can lead to variations in their hematological indices, biochemical parameters, and certain clinical parameters (35). This finding further confirms that in the complex physiological process of adapting to high-altitude environments, there may be complex and subtle interactions between genetic factors and environmental factors, jointly influencing the human body’s adaptability and physiological performance in high-altitude environments.

**Figure 4:**
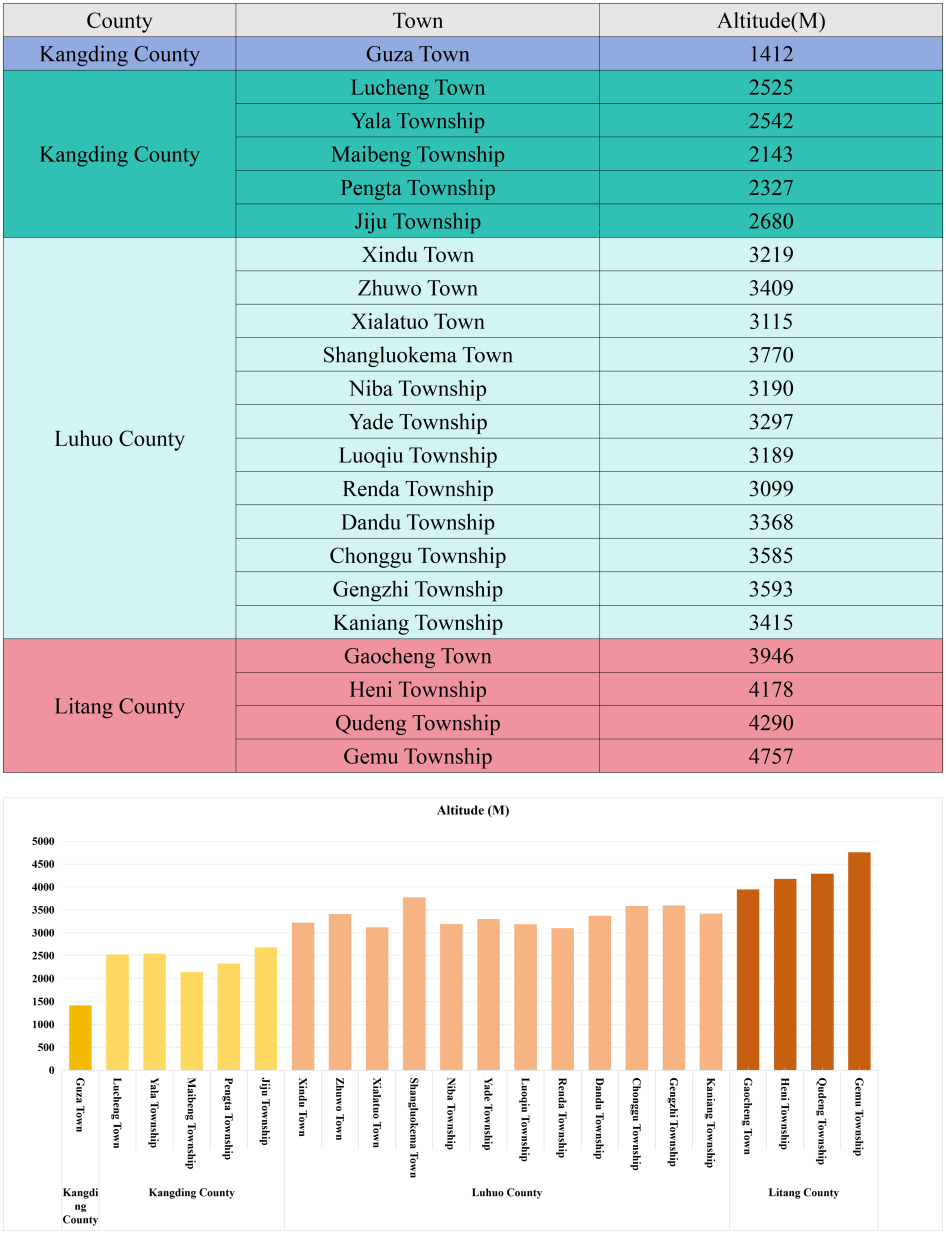
Table of altitudes in the study area.

HCT and HGB indicators in Litang County, RBC, HCT, and HGB levels in adult males were higher than in females across the other four regions. As altitude increased, these reference values also increased, consistent with previous research findings (36). In Litang County, the differences in HCT and HGB levels between sexes were statistically insignificant. This may be attributed to the extreme environmental conditions at very high altitudes, where the impact of altitude on HCT and HGB outweighs that of sex, leading to similar trends in HCT and HGB values between males and females (37). However, further studies are needed to verify this hypothesis. As shown in Figure 4, in the relatively lower-altitude regions of Guza Town and Lucheng Town, the CBC indicators for residents under 40 years of age were found to be lower than or at the lower end of the national reference intervals. As age increased, the CBC reference values increased, with RBC count, HCT, and HGB levels in individuals over 60 years of age significantly exceeding the national reference intervals. This further illustrates that the CBC differences between residents of high-altitude and low-altitude plain regions are not entirely innate but closely related to long-term exposure to chronic hypoxic conditions, reflecting the body’s adaptation to extreme environmental conditions (38, 39). Some studies have even found that, in order to adapt to the hypoxic environment at high altitudes and thus reduce the oxygen consumption of the fetus in the mother’s body, the birth weight of newborns in high-altitude areas is lower than that of newborns in low-altitude areas (40). Additionally, this difference in weight will persist throughout childhood and adolescence (41).

White blood cells (WBCs) are integral to the immune system, and their levels increase in response to infections, injuries, and other stress conditions as a part of the body’s mechanism (42). Our results showed a positive correlation between altitude and WBC counts for males and females. This may be due to the harsh environmental conditions at high altitudes, such as lower oxygen levels, low temperatures, and stronger ultraviolet radiation, which likely trigger a stress response (43). In response, the body releases cytokines and hormones such as adrenaline and cortisol, stimulating hematopoietic stem cells in the bone marrow and increasing WBC production. This process can potentially lead to local or systemic inflammatory responses activating the immune system, prompting WBCs to accumulate at sites of inflammation and stimulate the bone marrow to produce more WBCs. Furthermore, hypoxia may affect the function of vascular endothelial cells by increasing their permeability (44), facilitating the migration from the bloodstream into tissues, thereby further increasing WBC counts. Populations living at high altitudes may undergo adaptive changes in their immune systems, improving their ability to resist pathogens and cope with hypoxia by altering the activity and number of WBCs. The results indicated a positive correlation between age and the WBC reference values (45). For individuals aged < 40 years, the WBC reference values were below the national reference interval. In the 40–59 age group, WBC levels remained mostly at the lower end of the national reference intervals. However, in two relatively high-altitude regions, the WBC reference values for individuals over 60 years of age exceeded the national reference levels. These changes further support the concept of “genetic adaptation” and “natural acclimatization” among high-altitude residents, suggesting that their immune systems gradually adapt to hypoxic conditions over time, supporting survival in such environments (46–48).

Platelets (PLT) are a crucial component of the blood and are essential for hemostasis and thrombosis. At high altitudes, the body may adjust blood volume and viscosity to compensate for lower oxygen levels, which can indirectly affect platelet circulation and lifespan, thereby influencing platelet count (49). Prolonged exposure to high-altitude conditions can also lead to a chronic inflammatory and stress response, potentially activating platelet production and regulating their function, reflected in changes in PLT counts (50). However, this increase may also present potential risks, such as a heightened likelihood of thrombosis and cardiovascular incidents (51), necessitating further research to clarify the specific mechanisms and their broader impact on human health. This study indicates that platelet counts in high-altitude populations generally show an increasing trend. This phenomenon is likely related to enhanced bone marrow stimulation induced by the hypoxic environment, changes in hemodynamics, and the activation of stress responses within the body. Under hypoxic conditions, upregulation of hypoxia-inducible factor 1-alpha (HIF-1α) stimulates megakaryocytes in the bone marrow to produce more platelets, leading to increased platelet counts (52, 53). PLT level also exhibited similar patterns to WBC, with reference values for those under 40 years of age and in the 40–59 age group, primarily at the lower end of the national reference intervals. In the two relatively low-altitude areas, PLT reference values for individuals aged >60 years were mostly at the higher end of the national reference intervals. However, in the two relatively high-altitude areas, PLT reference values for individuals over 60 years generally exceeded the national reference levels. In addition to the factor of altitude, previous studies have also found that senescent hematopoietic stem cells can give rise to a new direct platelet differentiation pathway that is parallel to the classical pathway, and the generation through this new pathway will lead to an increase in the number of platelets with high activity (54). When conducting an in-depth exploration of platelets, the traditional view generally holds that the platelet count in men is lower than that in women (55, 56). However, this study shows that in regions at the same altitude, the platelet count in men is higher than that in women. After rigorously checking the experimental procedures and comprehensively eliminating potential errors, the phenomenon that the platelet values in women are lower than those in men in the Western Sichuan Plateau region is analyzed in depth from the following aspects: (1) Hormonal regulation: The hormonal levels in the human body will be affected and fluctuate in the plateau environment (57). Estrogen receptors (ERα/ERβ) are present in megakaryocytes. Estrogen inhibits the differentiation of megakaryocytes into platelets by down-regulating transcription factors such as GATA-1 and NF-E2 (58), and also reduces the reactivity of megakaryocytes to thrombopoietin (TPO), thereby reducing platelet release (59). (2) Genetic factors: During embryonic development, one of the two X chromosomes in female cells will randomly inactivate to form a Barr body (60), which may lead to incomplete expression of the GATA1 gene and affect platelet production (61). In the Western Sichuan Plateau, complex environmental factors may enhance the influence of genetic factors on platelet production. For example, environmental stresses such as plateau radiation may increase the probability of abnormal expression of the GATA1 gene during the inactivation of the X chromosome, thereby reducing the platelet production in women. (3) Environmental factors: The hypoxic environment (decreased PO₂) in the Western Sichuan Plateau will change the bone marrow hematopoietic microenvironment through the HIF-1α signaling pathway, inhibit the maturation of megakaryocytes, and reduce platelet production (62). Moreover, the strong ultraviolet rays in this area will damage the bone marrow hematopoietic stem cells through oxidative stress and interfere with platelet production (63). In addition, the different living and working habits between men and women may make women more vulnerable to the negative impacts of the environment.

By collecting and analyzing the blood routine indices of healthy people at different altitudes in the Western Sichuan Plateau, this study established the blood routine reference intervals for healthy adult residents in these areas. Meanwhile, the study also revealed some adaptive changes that occur in the human body when dealing with the complex high-altitude environment. These changes have a significant impact on the overall health status (64, 65). In addition, we found that the reference intervals of the complete blood count (CBC) vary depending on altitude, gender, and age, which reflects the comprehensive influence of the high-altitude environment on the human body (18). Moreover, the involvement of factors such as different living habits and genetic genes can also lead to changes in the complete blood count (CBC).

### Limitations of the study

**Figure 5:**
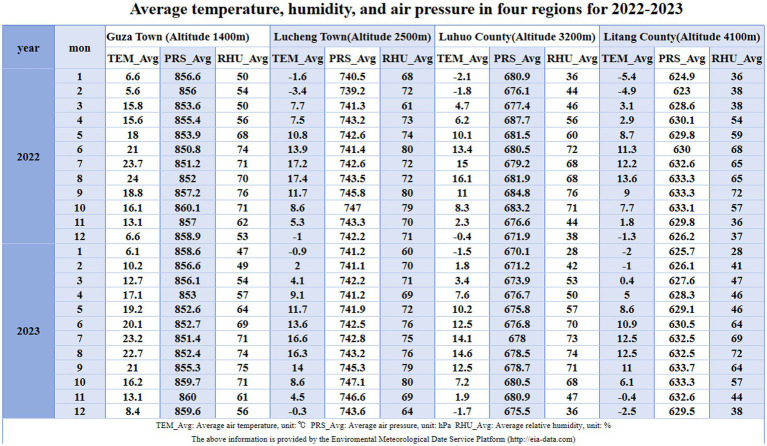
Table of average pressure, temperature, and humidity for 2022–2023 across four regions.

This study has successfully revealed the key changes in the blood routine indices of populations at different altitudes in the Western Sichuan Plateau and established reference intervals, providing important evidence for the research on high-altitude environments and human physiological adaptation. However, due to research limitations, potential factors like individual lifestyles, dietary structures, genetic differences, and blood collection seasons could not be explored deeply. The specific data are shown in Figure 5. Future research could expand sample coverage, incorporate more factors, analyze their interactions, improve the theoretical system, offer more comprehensive and accurate scientific support for the health of high-altitude residents, and propel related fields to new heights.

## Conclusion

Through collecting and analyzing the blood routine indices of healthy people at different altitudes in the Western Sichuan Plateau, this study has successfully established the reference intervals for the blood routine of healthy adult residents in this region. The study has clarified the adaptive changes of the human body in high-altitude environments. That is, as the altitude increases and the oxygen content decreases, the levels of white blood cells (WBC), red blood cells (RBC), hemoglobin (HGB), hematocrit (HCT), and platelets (PLT) increase. Moreover, the reference intervals for the complete blood count are affected by altitude, gender, and age. These achievements have laid the foundation for the prevention and diagnosis of related diseases among residents in the Western Sichuan Plateau, enhanced the understanding of how high-altitude environments affect the function of the immune system, and are conducive to optimizing public health strategies for people in high-altitude areas. In the future, it is expected that through continuous research on the differences in complete blood count parameters among adult populations in different altitude areas, more accurate diagnostic criteria can be provided for medical institutions in high-altitude regions, the medical process can be further optimized, the diagnostic accuracy can be improved, the health assessment of local residents can be strengthened, and the medical and health undertakings in high-altitude areas can be comprehensively promoted.

## Data Availability

The datasets presented in this study can be found in online repositories. The names of the repository/repositories and accession number(s) can be found in the article/Supplementary material.
