# Supplementary material for: Establishment of reference intervals for complete blood count in healthy adults at different altitudes on the Western Sichuan Plateau
Source: Front Med (Lausanne). 2025 May 21;12:1586778. doi: 10.3389/fmed.2025.1586778 (PMC12134580; doi:10.3389/fmed.2025.1586778)
Supplement: Supplementary file 1 [file Data_Sheet_1.PDF]

Average temperature, humidity, and atmospheric pressure in four regions for 2022-2023

year	mon	Guzan Town (Altitude 1400m)			Lucheng Town(Altitude 2500m)			Luhuo County(Altitude 3200m)			Litang County(Altitude 4100m)		
		TEM_Avg	PRS_Avg	RHU_Avg	TEM_Avg	PRS_Avg	RHU_Avg	TEM_Avg	PRS_Avg	RHU_Avg	TEM_Avg	PRS_Avg	RHU_Avg
2022	1	6.6	856.6	50	-1.6	740.5	68	-2.1	680.9	36	-5.4	624.9	36
	2	5.6	856	54	-3.4	739.2	72	-1.8	676.1	44	-4.9	623	38
	3	15.8	853.6	50	7.7	741.3	61	4.7	677.4	46	3.1	628.6	38
	4	15.6	855.4	56	7.5	743.2	73	6.2	687.7	56	2.9	630.1	54
	5	18	853.9	68	10.8	742.6	74	10.1	681.5	60	8.7	629.8	59
	6	21	850.8	74	13.9	741.4	80	13.4	680.5	72	11.3	630	68
	7	23.7	851.2	71	17.2	742.6	72	15	679.2	68	12.2	632.6	65
	8	24	852	70	17.4	743.5	72	16.1	681.9	68	13.6	633.3	65
	9	18.8	857.2	76	11.7	745.8	80	11	684.8	76	9	633.3	72
	10	16.1	860.1	71	8.6	747	79	8.3	683.2	71	7.7	633.1	57
	11	13.1	857	62	5.3	743.3	70	2.3	676.6	44	1.8	629.8	36
	12	6.6	858.9	53	-1	742.2	71	-0.4	671.9	38	-1.3	626.2	37
2023	1	6.1	858.6	47	-0.9	741.2	60	-1.5	670.1	28	-2	625.7	28
	2	10.2	856.6	49	2	741.1	70	1.8	671.2	42	-1	626.1	41
	3	12.7	856.1	54	4.1	742.2	71	3.4	673.9	53	0.4	627.6	47
	4	17.1	853	57	9.1	741.2	69	7.6	676.7	50	5	628.3	46
	5	19.2	852.6	64	11.7	741.9	72	10.2	675.8	57	8.6	629.1	46
	6	20.1	852.7	69	13.6	742.5	76	12.5	676.8	70	10.9	630.5	64
	7	23.2	851.4	71	16.6	742.8	75	14.1	678	73	12.5	632.5	69
	8	22.7	852.4	74	16.3	743.2	76	14.6	678.5	74	12.5	632.5	72
	9	21	855.3	75	14	745.3	79	12.5	678.7	71	11	633.7	64
	10	16.2	859.7	71	8.6	747.1	80	7.2	680.5	68	6.1	633.3	57
	11	13.1	860	61	4.5	746.6	69	1.9	680.9	47	-0.4	632.6	44
	12	8.4	859.6	56	-0.3	743.6	64	-1.7	675.5	36	-2.5	629.5	38

TEM_Avg: Average air temperature, unit: ℃ PRS_Avg: Average air pressure, unit: hPa RHU_Avg: Average relative humidity, unit: %

The above information is provided by the Environmental Meteorological Data Service Platform (http://eia-data.com/).
