# Supplementary material for: Establishment of reference intervals for complete blood count in healthy adults at different altitudes on the Western Sichuan Plateau
Source: Front Med (Lausanne). 2025 May 21;12:1586778. doi: 10.3389/fmed.2025.1586778 (PMC12134580; doi:10.3389/fmed.2025.1586778)
Supplement: Supplementary file 2 [file Data_Sheet_2.PDF]

County	Town	Altitude(M)
Kangding County	Guza Town	1412
Kangding County	Lucheng Town	2525
	Yala Township	2542
	Maibeng Township	2143
	Pengta Township	2327
	Jiju Township	2680
Luhuo County	Xindu Town	3219
	Zhuwo Town	3409
	Xialatuo Town	3115
	Shangluokema Town	3770
	Niba Township	3190
	Yade Township	3297
	Luoqiu Township	3189
	Renda Township	3099
	Dandu Township	3368
	Chonggu Township	3585
	Gengzhi Township	3593
	Kaniang Township	3415
Litang County	Gaocheng Town	3946
	Heni Township	4178
	Qudeng Township	4290
	Gemu Township	4757

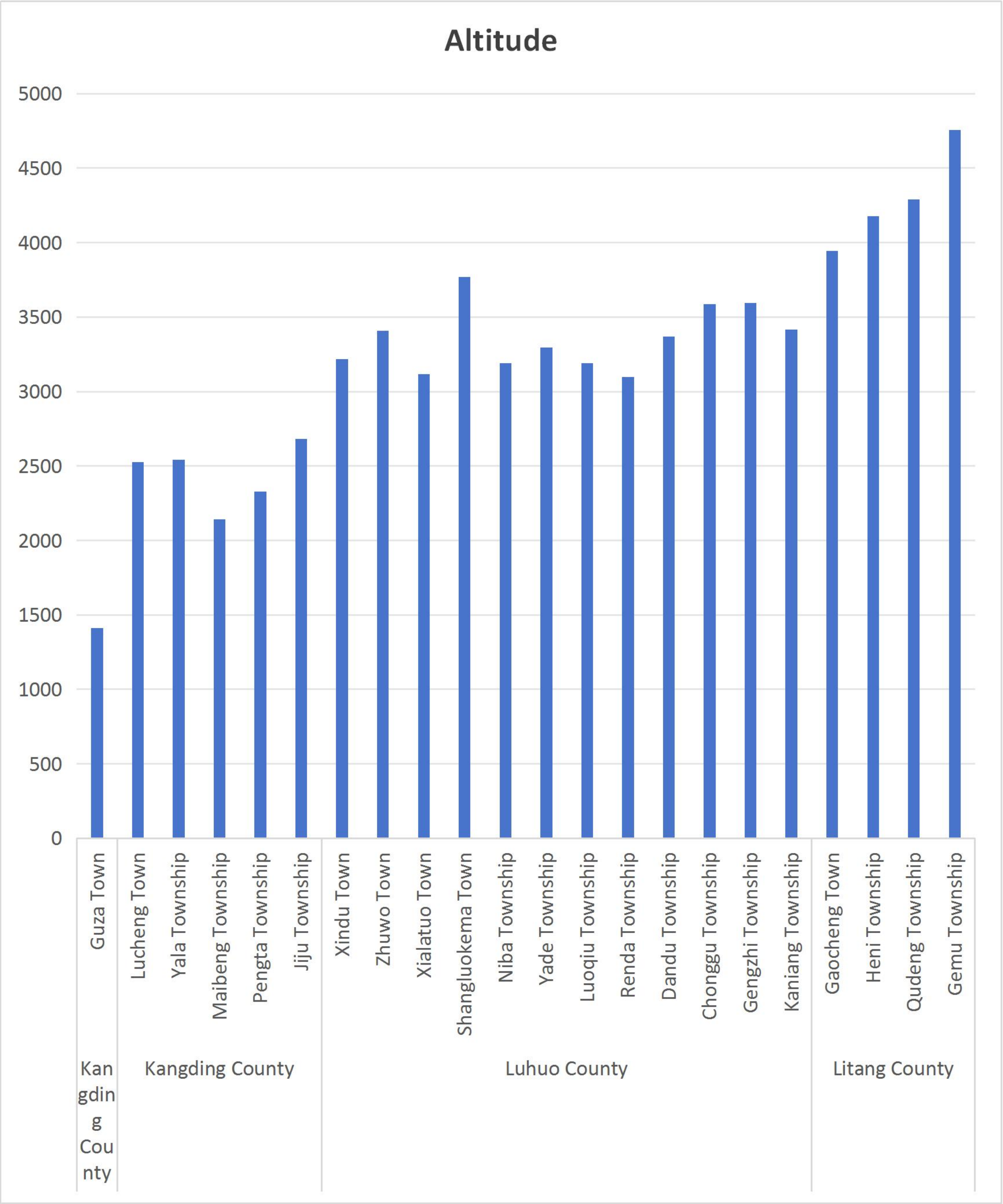
Altitude
5000
4500
4000
3500
3000
2500
2000
1500
1000
500
0
Guza Town
Lucheng Town
Yala Township
Maibeng Township
Pengta Township
Jiju Township
Xindu Town
Zhuwo Town
Xialatuo Town
Shangluokema Town
Niba Township
Yade Township
Luoqiu Township
Renda Township
Dandu Township
Chonggu Township
Gengzhi Township
Kaniang Township
Gaocheng Town
Heni Township
Qudeng Township
Gemu Township
Kangding County
Kangding County
Luhuo County
Litang County
