# Supplementary material for: Establishment of reference intervals for complete blood count in healthy adults at different altitudes on the Western Sichuan Plateau
Source: Front Med (Lausanne). 2025 May 21;12:1586778. doi: 10.3389/fmed.2025.1586778 (PMC12134580; doi:10.3389/fmed.2025.1586778)
Supplement: Supplementary file 3 [file Data_Sheet_3.PDF]

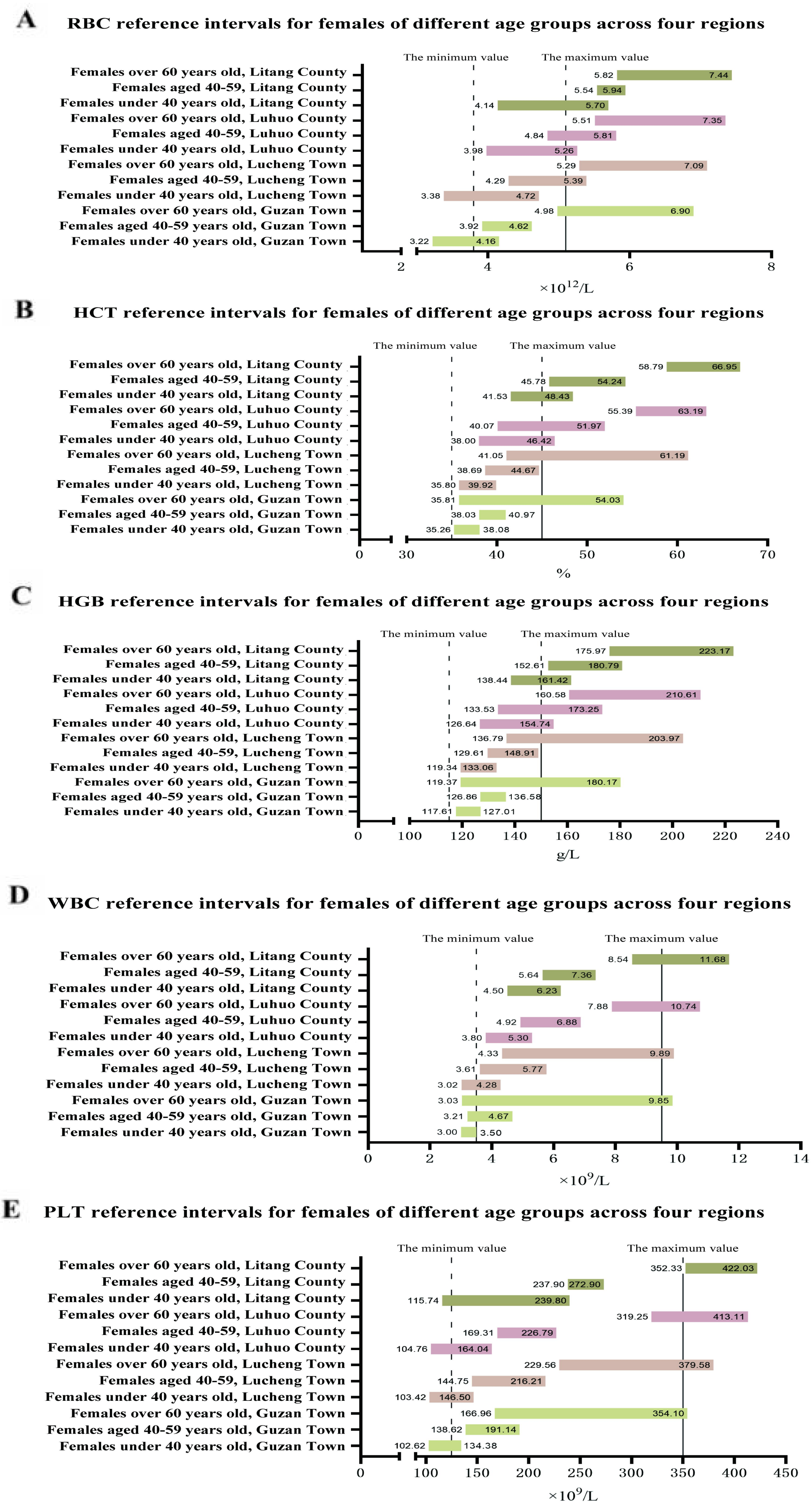
A
RBC reference intervals for females of different age groups across four regions
The minimum value
The maximum value
Females over 60 years old, Litang County
Females aged 40-59, Litang County
Females under 40 years old, Litang County
Females over 60 years old, Luhuo County
Females aged 40-59, Luhuo County
Females under 40 years old, Luhuo County
Females over 60 years old, Lucheng Town
Females aged 40-59, Lucheng Town
Females under 40 years old, Lucheng Town
Females over 60 years old, Guzan Town
Females aged 40-59 years old, Guzan Town
Females under 40 years old, Guzan Town
5.82 7.44
5.54 5.94
4.14 5.70
5.51 7.35
4.84 5.81
3.98 5.26
5.29 7.09
4.29 5.39
3.38 4.72
4.98 6.90
3.92 4.62
3.22 4.16
2 4 6 8
×10^12/L
B
HCT reference intervals for females of different age groups across four regions
The minimum value
The maximum value
58.79 66.95
45.78 54.24
41.53 48.43
55.39 63.19
40.07 51.97
38.00 46.42
41.05 61.19
38.69 44.67
35.80 39.92
35.81 54.03
38.03 40.97
35.26 38.08
0 30 40 50 60 70
%
C
HGB reference intervals for females of different age groups across four regions
The minimum value
The maximum value
175.97 223.17
152.61 180.79
138.44 161.42
160.58 210.61
133.53 173.25
126.64 154.74
136.79 203.97
129.61 148.91
119.34 133.06
119.37 180.17
126.86 136.58
117.61 127.01
0 100 120 140 160 180 200 220 240
g/L
D
WBC reference intervals for females of different age groups across four regions
The minimum value
The maximum value
8.54 11.68
5.64 7.36
4.50 6.23
7.88 10.74
4.92 6.88
3.80 5.30
4.33 9.89
3.61 5.77
3.02 4.28
3.03 9.85
3.21 4.67
3.00 3.50
0 2 4 6 8 10 12 14
×10^9/L
E
PLT reference intervals for females of different age groups across four regions
The minimum value
The maximum value
352.33 422.03
237.90 272.90
115.74 239.80
319.25 413.11
169.31 226.79
104.76 164.04
229.56 379.58
144.75 216.21
103.42 146.50
166.96 354.10
138.62 191.14
102.62 134.38
0 100 150 200 250 300 350 400 450
×10^9/L
