# Supplementary material for: Establishment of reference intervals for complete blood count in healthy adults at different altitudes on the Western Sichuan Plateau
Source: Front Med (Lausanne). 2025 May 21;12:1586778. doi: 10.3389/fmed.2025.1586778 (PMC12134580; doi:10.3389/fmed.2025.1586778)
Supplement: Supplementary file 4 [file Data_Sheet_4.PDF]

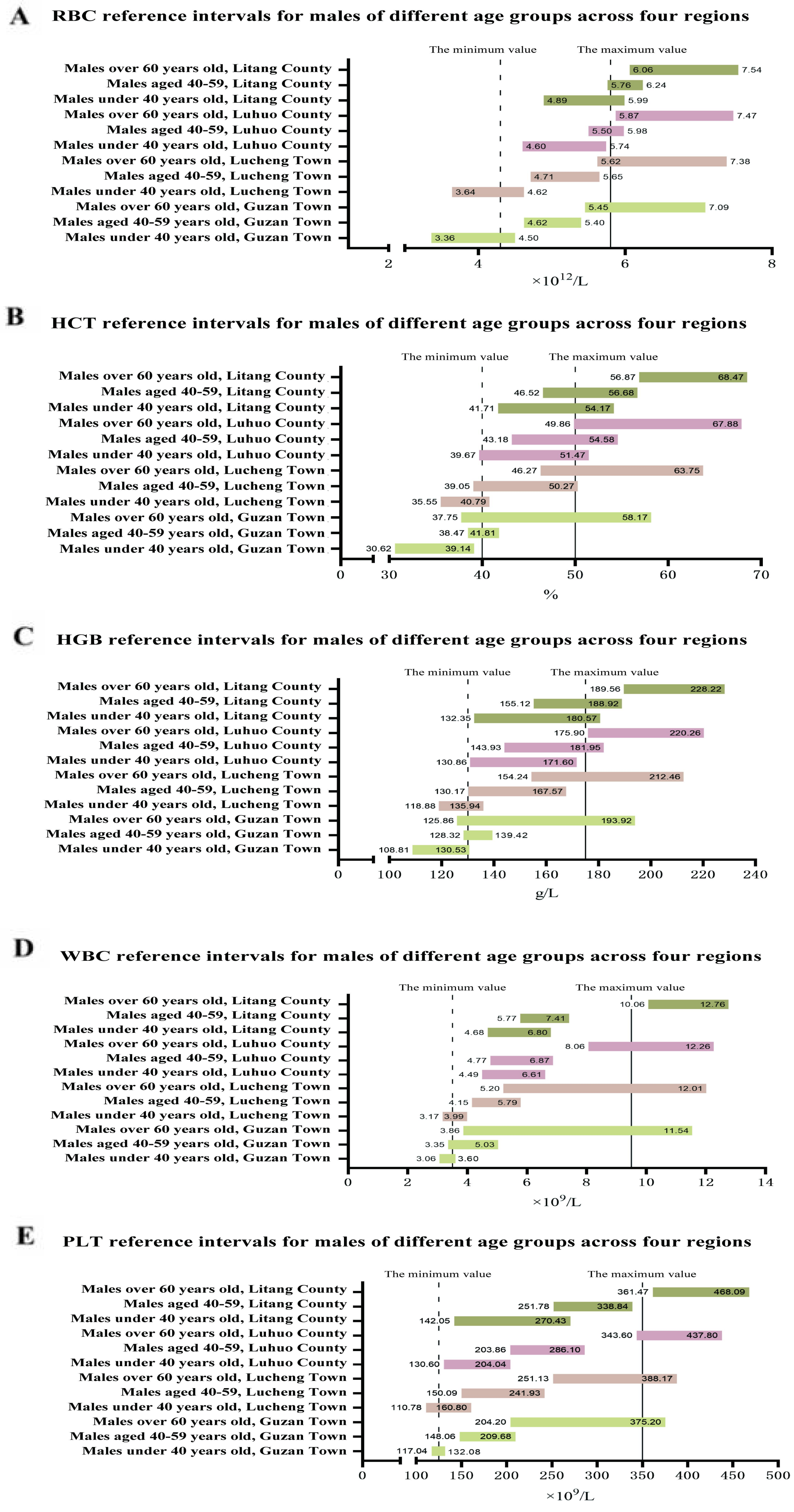
A
RBC reference intervals for males of different age groups across four regions
The minimum value
The maximum value
Males over 60 years old, Litang County
Males aged 40-59, Litang County
Males under 40 years old, Litang County
Males over 60 years old, Luhuo County
Males aged 40-59, Luhuo County
Males under 40 years old, Luhuo County
Males over 60 years old, Lucheng Town
Males aged 40-59, Lucheng Town
Males under 40 years old, Lucheng Town
Males over 60 years old, Guzan Town
Males aged 40-59 years old, Guzan Town
Males under 40 years old, Guzan Town
6.06 7.54
5.76 6.24
4.89 5.99
5.87 7.47
5.50 5.98
4.60 5.74
5.62 7.38
4.71 5.65
3.64 4.62
5.45 7.09
4.62 5.40
3.36 4.50
2 4 6 8
×10^12/L
B
HCT reference intervals for males of different age groups across four regions
The minimum value
The maximum value
Males over 60 years old, Litang County
Males aged 40-59, Litang County
Males under 40 years old, Litang County
Males over 60 years old, Luhuo County
Males aged 40-59, Luhuo County
Males under 40 years old, Luhuo County
Males over 60 years old, Lucheng Town
Males aged 40-59, Lucheng Town
Males under 40 years old, Lucheng Town
Males over 60 years old, Guzan Town
Males aged 40-59 years old, Guzan Town
Males under 40 years old, Guzan Town
56.87 68.47
46.52 56.68
41.71 54.17
49.86 67.88
43.18 54.58
39.67 51.47
46.27 63.75
39.05 50.27
35.55 40.79
37.75 58.17
38.47 41.81
30.62 39.14
0 30 40 50 60 70
%
C
HGB reference intervals for males of different age groups across four regions
The minimum value
The maximum value
Males over 60 years old, Litang County
Males aged 40-59, Litang County
Males under 40 years old, Litang County
Males over 60 years old, Luhuo County
Males aged 40-59, Luhuo County
Males under 40 years old, Luhuo County
Males over 60 years old, Lucheng Town
Males aged 40-59, Lucheng Town
Males under 40 years old, Lucheng Town
Males over 60 years old, Guzan Town
Males aged 40-59 years old, Guzan Town
Males under 40 years old, Guzan Town
189.56 228.22
155.12 188.92
132.35 180.57
175.90 220.26
143.93 181.95
130.86 171.60
154.24 212.46
130.17 167.57
118.88 135.94
125.86 193.92
128.32 139.42
108.81 130.53
0 100 120 140 160 180 200 220 240
g/L
D
WBC reference intervals for males of different age groups across four regions
The minimum value
The maximum value
Males over 60 years old, Litang County
Males aged 40-59, Litang County
Males under 40 years old, Litang County
Males over 60 years old, Luhuo County
Males aged 40-59, Luhuo County
Males under 40 years old, Luhuo County
Males over 60 years old, Lucheng Town
Males aged 40-59, Lucheng Town
Males under 40 years old, Lucheng Town
Males over 60 years old, Guzan Town
Males aged 40-59 years old, Guzan Town
Males under 40 years old, Guzan Town
10.06 12.76
5.77 7.41
4.68 6.80
8.06 12.26
4.77 6.87
4.49 6.61
5.20 12.01
4.15 5.79
3.17 3.99
3.66 11.54
3.35 5.03
3.06 3.60
0 2 4 6 8 10 12 14
×10^9/L
E
PLT reference intervals for males of different age groups across four regions
The minimum value
The maximum value
Males over 60 years old, Litang County
Males aged 40-59, Litang County
Males under 40 years old, Litang County
Males over 60 years old, Luhuo County
Males aged 40-59, Luhuo County
Males under 40 years old, Luhuo County
Males over 60 years old, Lucheng Town
Males aged 40-59, Lucheng Town
Males under 40 years old, Lucheng Town
Males over 60 years old, Guzan Town
Males aged 40-59 years old, Guzan Town
Males under 40 years old, Guzan Town
361.47 468.09
251.78 338.84
142.05 270.43
343.60 437.80
203.86 286.10
130.60 204.04
251.13 388.17
150.09 241.93
110.78 160.80
204.20 375.20
148.06 209.68
117.04 132.08
0 100 150 200 250 300 350 400 450 500
×10^9/L
