# Supplementary figures and images for: Establishment of reference intervals for complete blood count in healthy adults at different altitudes on the Western Sichuan Plateau
Source: Front Med (Lausanne). 2025 May 21;12:1586778. doi: 10.3389/fmed.2025.1586778 (PMC12134580; doi:10.3389/fmed.2025.1586778)

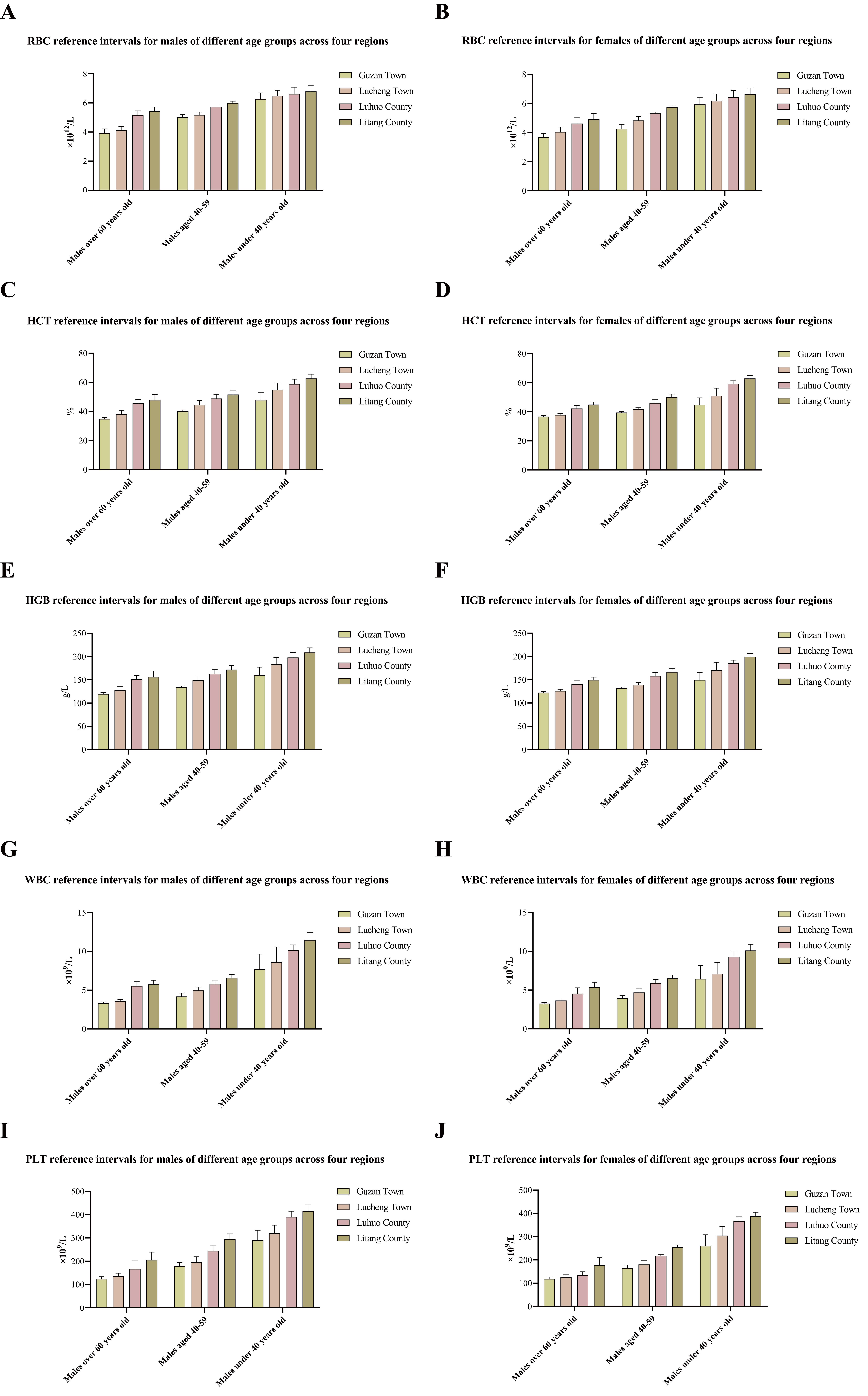

Supplement: Supplementary file 5 [file Image_1.PNG]
